# Patients with Irritable Bowel Syndrome Exhibit Aberrant Expression of Endogenous Retroviruses and SETDB1

**DOI:** 10.3390/cells14030196

**Published:** 2025-01-29

**Authors:** Pier-Angelo Tovo, Davide Giuseppe Ribaldone, Gian Paolo Caviglia, Cristina Calvi, Paola Montanari, Marco Tizzani, Demis Pitoni, Simone Frara, Elisa Tribocco, Stefano Gambarino, Marta Guariglia, Ilaria Galliano, Massimiliano Bergallo

**Affiliations:** 1 Department of Public Health and Pediatric Sciences, University of Turin, Piazza Polonia 94, 10126 Turin, Italy; pierangelo.tovo@unito.it; 2 Department of Medical Sciences, Division of Gastroenterology, University of Turin, 10123 Turin, Italy; davidegiuseppe.ribaldone@unito.it (D.G.R.); gianpaolo.caviglia@unito.it (G.P.C.); marco.tizzani91@gmail.com (M.T.); demis.pitoni@unito.it (D.P.); docfrara@gmail.com (S.F.); elisa.tribocco@edu.unito.it (E.T.); marta.guariglia@unito.it (M.G.); 3 Pediatric Laboratory, Department of Public Health and Pediatric Sciences, University of Turin, Regina Margherita Children’s Hospital, Piazza Polonia 94, 10126 Turin, Italy; cristina.calvi@unito.it (C.C.); paola.montanari@unito.it (P.M.); stefano.gambarino@unito.it (S.G.); massimiliano.bergallo@unito.it (M.B.)

**Keywords:** irritable bowel syndrome, human endogenous retroviruses, TRIM28, SETDB1, pathogenesis

## Abstract

Irritable bowel syndrome (IBS) is a common disease, whose etiopathogenesis is poorly understood. Human endogenous retroviruses (HERVs) originate from ancient infections of germinal cells and represent 8% of our DNA. Most HERVs have become defective due to the accumulated mutations; some can, however, still be activated, and their altered expressions have been associated with a number of chronic inflammatory and immune-mediated disorders, including gastrointestinal diseases. Retroviral transcription is modulated by TRIM28 and SETDB1, which also participate in the regulation of epigenetic mechanisms and in shaping the immune system. Expressions of HERVs and TRIM28/SETDB1 have not been investigated in patients affected by IBS. Using a PCR real-time Taqman amplification assay, we explored the RNA levels of HERV-H-pol, HERV-K-pol, and HERV-W-pol; syncytin 1 (SYN1), SYN2, and HERV-W-env; and TRIM28 and SETDB1 in the peripheral blood of 37 IBS patients and healthy controls (HCs) of similar age. The transcript levels were higher in IBS patients than in HCs for all HERVs except for HERV-W-pol, with significant *p*-values for HERV-H-pol, HERV-K-pol, and SYN1 and borderline *p*-values for SYN2 and HERV-W-env. The RNA levels of SETDB1 were significantly enhanced in IBS patients, while those of TRIM28 were in the normal range. Patients with severe disease had significant upregulation of SETDB1 compared to those with mild or moderate symptoms. These findings suggest that overexpression of HERVs and SETDB1 may contribute to the development of IBS and open the way to innovative therapeutic strategies.

## 1. Introduction

Irritable bowel syndrome (IBS) is a persistent gastrointestinal disorder characterized by recurrent abdominal pain with altered bowel habits [1]. It affects approximately 5–10% of the global population [2,3], significantly impacting quality of life and posing a substantial economic burden due to healthcare costs and reduced productivity [4]. There are distinct clinical types of IBS. Among these, the diarrhea-predominant IBS (IBS-D) is one of the most common. It is characterized by frequent loose stools and urgency, in addition to abdominal pain [5].

The etiopathogenesis of IBS is poorly understood [6]. Historically, it was classified as a functional gastrointestinal disorder without organic abnormalities [7]. Recent advances have, however, led to its reclassification as a disorder of gut–brain interaction (DGBI) [8,9,10]. This paradigm shift acknowledges the complex reciprocal communications between the gastrointestinal tract and the central nervous system, involving neural, hormonal, and immunological pathways [9,10,11]. Contributing factors include altered gastrointestinal motility, visceral hypersensitivity, psychosocial stress, dysbiosis of the gut microbiota, intestinal inflammation, and immune activation [12,13,14].

Human endogenous retroviruses (HERVs) derive from ancestral infections of germinal cells millions of years ago. Subsequently, they were transmitted to every future generation through Mendelian inheritance [15]. They constitute approximately 8% of human DNA [16]. HERVs maintain their retroviral structure with three principal genes, namely group-associated antigens (gag), polymerase (pol), and envelope (env), flanked between two regulatory long terminal repeats (LTRs) [16]. Due to the accumulated mutations, depletions, and duplications, most retroviral elements are no longer able to produce infectious particles. Some, however, retain the ability to be transcribed, and a few encode proteins that are co-opted for essential physiological functions during intrauterine life, such as syncytin (SYN) 1 [17] and syncytin 2 (SYN 2) [18]. These participate in the formation of the placenta, in particular in the syncytiotrophoblast layer, and possess potent immunosuppressive activities that contribute to materno-fetal tolerance. HERVs can influence the host gene expression [19,20] and shape the immune system through several mechanisms, such as activation of innate immunity via toll-like receptors (TLRs), induction of pro-inflammatory cytokines, differentiation of B and T cells, and production of specific immune responses against tissue antigens or through molecular mimicry [20,21,22,23]. An extensive body of literature documents the association between enhanced HERV expressions and immune-mediated diseases [20,21,22,23,24,25,26], including gastrointestinal disorders, such as inflammatory bowel disease [27], food allergy or food-protein-induced enterocolitis syndrome (FPIES) [28], and celiac disease [29].

HERV expression is tightly regulated by epigenetic mechanisms via DNA methylation and histone tail modifications. Tripartite motif-containing 28 (TRIM28, also known as KAP1 or TIF1β) and SET domain bifurcated histone lysine methyltransferase 1 (SETDB1, also known as ESET) are transcriptional corepressors acting in concert with Krüppel-associated box domain zinc finger proteins (KRAB-ZNFs) to induce DNA methylation and heterochromatin formation. TRIM28 and SETDB1 play pivotal roles in silencing HERV transcription through epigenetic processes [30,31,32]. They are also directly implicated in the regulation of innate and adaptive immune responses [33,34,35].

Given the role of HERVs and TRIM28/SETDB1 in inflammation and immune activation, it is plausible that they may contribute to the pathophysiology of IBS. Their dysregulation could stimulate abnormal immune reactivity in the gut, leading to low-grade inflammation and altered gut motility and sensitivity [12,13,14]. To date, no studies have, however, investigated the transcription levels of HERVs and TRIM28/SETDB1 in patients with IBS. Exploring these factors could provide novel insights into the mechanisms responsible for the development of the disease and identify potential new therapeutic targets. Therefore, we assessed the transcriptional levels of HERV-H-pol, HERV-K-pol, and HERV-W-pol, the three most studied families of retroviruses; syncytin-1 (SYN1) and syncytin-2 (SYN2), whose proteins strongly influence the immune response [36,37], and HERV-W-env, a molecule able to trigger vigorous inflammatory reactions [21,25]; and TRIM28 and SETDB1 in the peripheral blood of IBS-D patients and healthy controls (HCs) of similar age.

## 2. Material and Methods

### 2.1. Study Populations

The enrollment was based on the diagnosis of IBS subtype D according to the Rome IV criteria [5]. The exclusion criteria were as follows: age <18 years or >65 years; gastrointestinal surgery; inflammatory bowel disease; celiac disease; uncontrolled thyroid diseases; diverticular disease; small bowel bacterial overgrowth (SIBO); colorectal cancer; clinically relevant organic, systemic, or metabolic diseases; use of medications that alter intestinal function; use of antibiotics or probiotics; and pregnancy or breastfeeding.

Symptom severity in IBS was defined using the Irritable Bowel Syndrome Severity Scoring System (IBS-SSS) questionnaire [38], which assesses five key factors: the presence and intensity of abdominal pain, the frequency of abdominal pain, the presence and severity of intestinal bloating, satisfaction with bowel habits, and the impact of symptoms on daily life. Each factor is scored on a scale from 0 to 100, resulting in a total score ranging from 0 to 500. Based on this score, IBS severity is categorized as mild (75–175), moderate (176–299), or severe (≥300), with scores below 75 being considered normal. Blood samples from IBS-D patients were obtained during usual laboratory analyses.

Healthy volunteers (regular blood donors) were the control group. Their blood samples were collected during donations. All denied ever having had any significant disease, including gastrointestinal disorders. They were chosen for age and gender comparable to those of the patients.

### 2.2. Total RNA Extraction

The procedures to measure the transcription levels of the target genes have previously been reported [26,27,28,29]. In particular, total RNA was extracted using the Maxwell automated extractor and the RNA Blood Kit (Promega, Madison, WI, USA), which includes a DNase treatment step to ensure the removal of any potential DNA contamination. To confirm the absence of DNA, RNA extracts were also directly amplified as a control step. The concentration and purity of the RNA were determined using UV spectrophotometry, measuring absorbance at 260 and 280 nm with a SimpliNano spectrophotometer (Biochrom US, Holliston, MA, USA). This step allowed the RNA’s quality and suitability for downstream applications to be verified. Once processed, the RNA samples were aliquoted and stored at −80 °C to maintain integrity until the final analysis.

### 2.3. Reverse Transcription

A total of 400 nanograms of RNA was reverse-transcribed in a 20 µL reaction mixture designed to maximize efficiency and accuracy in cDNA synthesis. The mixture contained 2 µL of 10× reaction buffer, 4.8 µL of 25 mM MgCl2 to optimize the enzyme activity, 2 µL of ImProm-II reverse transcriptase (Promega), and 1 µL of RNase inhibitor (20 U/µL) to protect the RNA from degradation. Additionally, 0.4 µL of 250 µM random hexamers (Promega) were included to prime the reverse transcription, along with 2 µL of 100 mM dNTP mix (Promega) to supply the necessary nucleotides for cDNA synthesis. The volume was brought to 20 µL with nuclease-free water to ensure the reaction proceeded under sterile and contamination-free conditions. The reverse transcription process was carried out using a GeneAmp PCR System 9700 Thermal Cycler (Applied Biosystems, Foster City, CA, USA) under a controlled program: an initial incubation at 25 °C for 5 min to facilitate primer binding, followed by 60 min at 42 °C to enable efficient enzyme activity and cDNA synthesis, and a final step at 70 °C for 15 min to inactivate the reverse transcriptase enzyme. After completion, the cDNA samples were stored at −20 °C to preserve their stability for subsequent analyses.

### 2.4. Transcription Levels of pol Genes of HERV-H, -K, and -W; env Genes of SYN1, SYN2, and HERV-W; and TRIM8/SETDB1 by a Real-Time PCR Assay

The relative expression (RE) levels of HERV-H-pol, HERV-K-pol, and HERV-W-pol; SYN1, SYN2, and HERV-W-env; and TRIM28/SETDB1 were quantified via the primers and probes detailed in Table 1.

Specifically, 40 ng of cDNA was amplified in a 20 µL reaction mixture optimized for efficient quantitative analysis. The mixture included 2.5 U of goTaq Master Mix (Promega), which provided the necessary DNA polymerase and buffer components, 1.25 mmol/L MgCl2 to enhance enzyme activity, 500 nM of specific primers designed to target the gene of interest, and 200 nM of specific fluorescent probes for real-time detection. The amplification reactions were carried out on a 96-well plate using a thermal cycler configured for real-time PCR. An initial denaturation at 95 °C for 10 min ensured complete separation of the cDNA strands and enzyme activation. This was followed by 45 cycles of denaturation at 95 °C for 15 s and annealing/extension at 60 °C for 1 min to optimize specificity and efficiency. Each sample was assessed in triplicate to ensure reproducibility and to minimize variability.

The RE of each transcript was calculated using the 2−∆∆Ct method [39]. GAPDH was chosen as the reference gene in all determinations, as it is one of the most stable among the reference genes and has already been used in our previous investigations [26,27,28,29]. After normalizing the PCR results of each target gene with GAPDH, these data were further calibrated with the median expression of the same gene from a pool of healthy controls. As Ct values were evaluated for all targets, we concluded that our method was robust for quantifying HERV and TRIM28/SETDB1 expressions.

### 2.5. Statistical Analysis

The Mann–Whitney test was employed to compare the RE of each target gene between patients affected by IBS-D and healthy controls. Statistical analyses were performed using Prism 7 software (GraphPad Software); *p* < 0.05 was the threshold of significance.

## 3. Results

### 3.1. Study Populations

A total of 37 patients affected by IBS-D and 95 healthy volunteers were studied. The demographic and clinical characteristics of the patients and HCs are reported in Table 1. The median age of IBS-D patients was similar to that of control subjects (*p* = 0.98), as was the percentage of males/females (Table 2).

### 3.2. Expression Levels of HERV-H-pol, HERV-K-pol, and HERV-W-pol in the Peripheral Blood of Patients with Diarrhea-Predominant Irritable Bowel Syndrome (IBS-D) and Healthy Controls (HCs)

**Figure 1:**
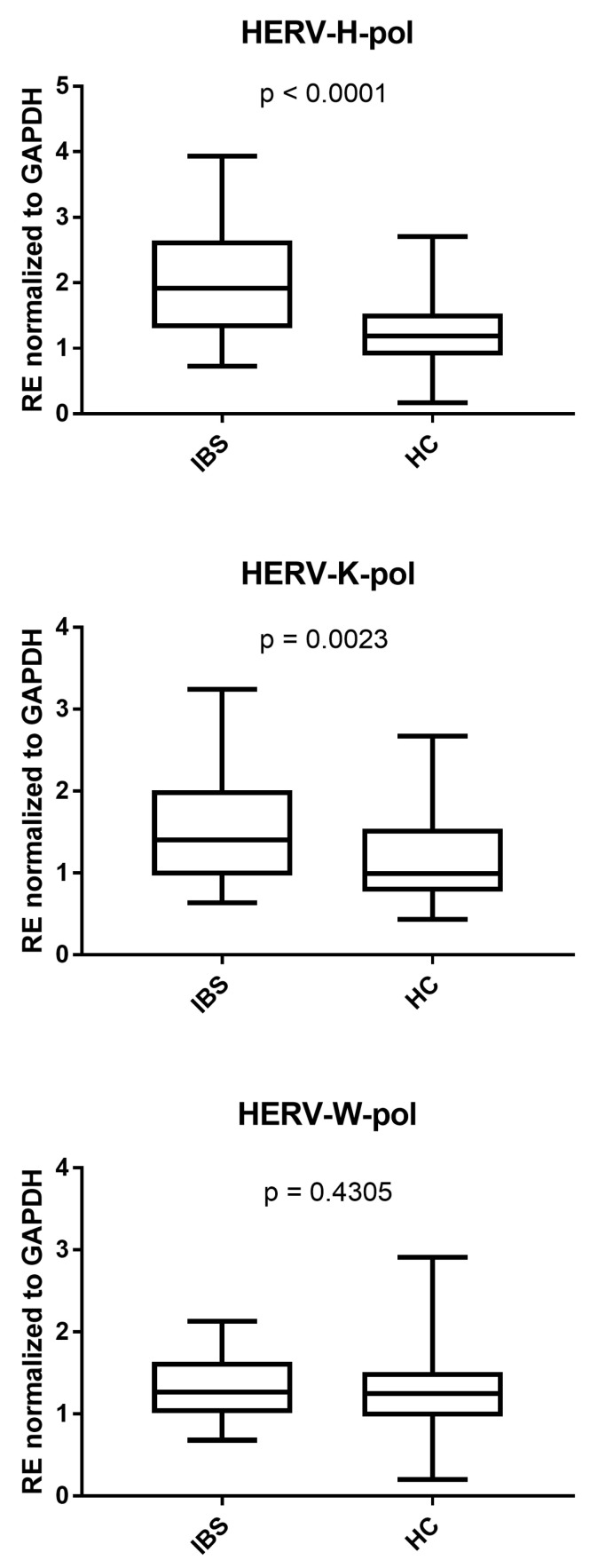
Transcriptional levels of HERV-H-pol, HERV-K-pol, and HERV-W-pol in the peripheral blood of 37 patients with diarrhea-predominant inflammatory bowel syndrome (IBS) and 95 healthy controls (HCs). RE: relative expression calculated using the 2−∆∆Ct method. Results are represented with whisker box plots: the boxes show the median and interquartile ranges 25–75%, and the whiskers indicate the minimum and maximum values. The *p*-values show the Mann–Whitney test results.

As reported in Figure 1, the RNA levels of pol genes of HERV-H and HERV-K were significantly higher in IBS-D patients than in HCs, while no difference emerged for HERV-W. The medians and IQR 25–75% were HERV-H-pol: IBS: 1.91, 1.33–2.63, HC: 1.19, 0.91–1.52; HERV-K-pol: IBS: 1.40, 0.98–1.96, HC: 0.99, 0.78–1.50; and HERV-W-pol: IBS: 1.27, 1.01–1.63, HC: 1.25, 0.98–1.50.

### 3.3. Transcription Levels of Syncytin 1, Syncytin 2, and HERV-W-env in the Peripheral Blood of Patients with Diarrhea-Predominant Irritable Bowel Syndrome (IBS) and Healthy Controls (HCs)

**Figure 2:**
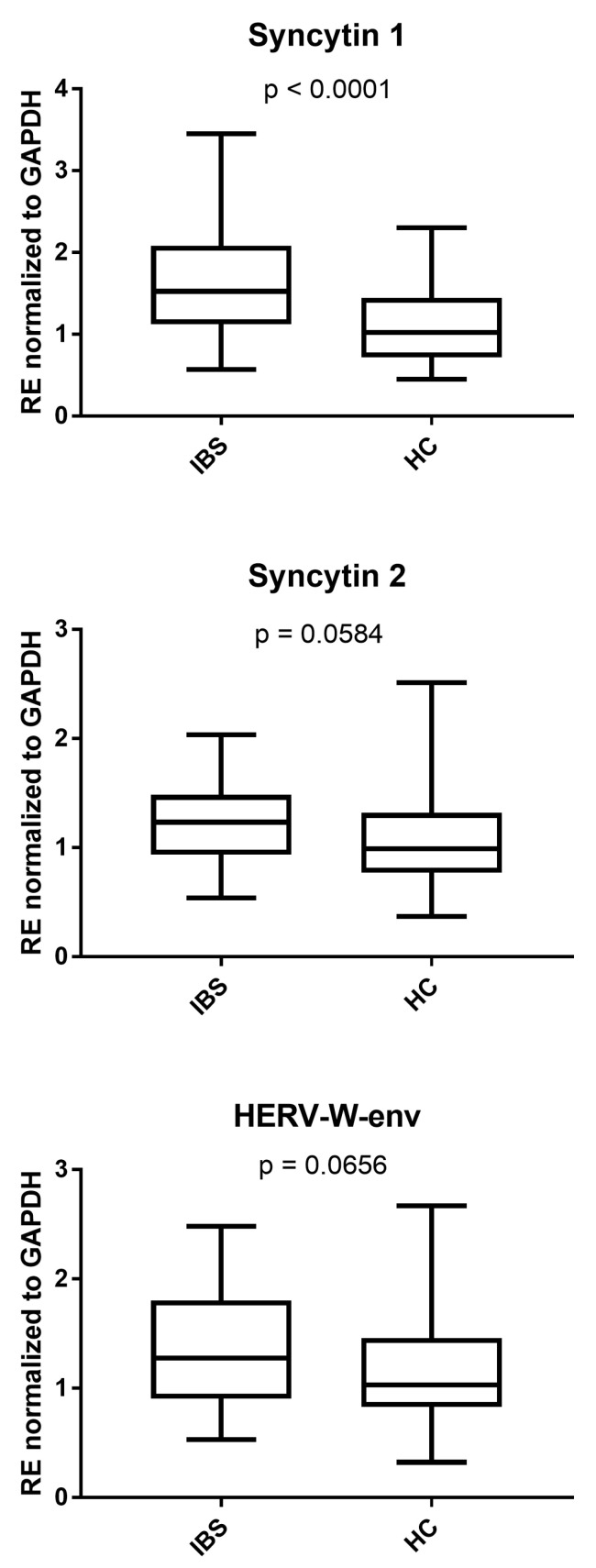
Transcriptional levels of syncytin 1, syncytin 2, and HERV-W-env in the peripheral blood of 37 patients with diarrhea-predominant irritable bowel syndrome (IBS) and 95 healthy controls (HCs). RE: relative expression calculated using the 2−∆∆Ct method. Results are represented with whisker box plots: the boxes show the median and interquartile ranges 25–75%, and the whiskers show the minimum and maximum values. The *p*-values show the Mann–Whitney test results.

The median values of SYN1 were significantly enhanced in IBS-D patients compared to HCs. Those of SYN2 and HERV-W-env were more increased, though at borderline *p*-values, in IBS-D patients compared to HCs (Figure 2). The medians and IQR 25–75% were syncytin 1: IBS: 1.53, 1.27–2.08, HC: 1.02, 0.72–1.43; syncytin 2: IBS: 1.23, 0.96–1.48, HC: 0.99, 0.78–1.32; and HERV-W-env: IBS: 1.28, 0.94–1.80, HC: 1.03, 0.83–1.46.

### 3.4. Transcriptional Levels of TRIM28 and SETDB1 in Patients with Diarrhea-Predominant Irritable Bowel Syndrome (IBS-D) and Healthy Controls (HCs)

**Figure 3:**
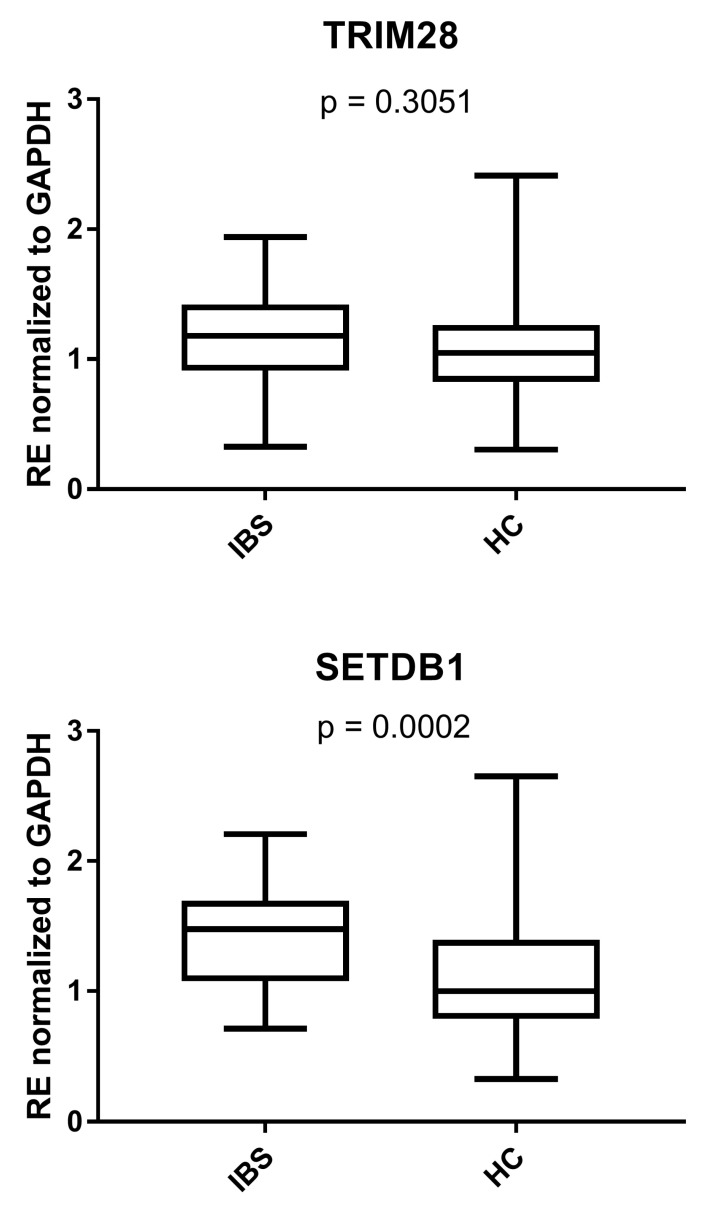
Expression levels of TRIM28 and SETDB1 in the peripheral blood of 37 patients with diarrhea-predominant irritable bowel syndrome (IBS) and 95 healthy controls (HCs). RE: relative expression calculated using the 2−∆∆Ct method. Results are represented with whisker box plots: the boxes show the median and interquartile ranges 25–75%, and the whiskers show the minimum and maximum values. The *p*-values show the Mann–Whitney test results.

As detailed in Figure 3, the median RNA levels of SETDB1 were significantly higher in IBS-D patients than in HCs, while those of TRIM28 were comparable between the two groups. The medians and IQR 25–75% were TRIM28: IBS: 1.18, 0.92–1.41, HC: 1.05, 0.83–1.26 and SETDB1: IBS: 1.29, 1.07–1.69, HC: 1.00, 0.80–1.39.

### 3.5. Expressions of HERVs, TRIM28, and SETDB1 in Patients with Diarrhea-Predominant IBS According to Disease Activity

**Figure 4:**
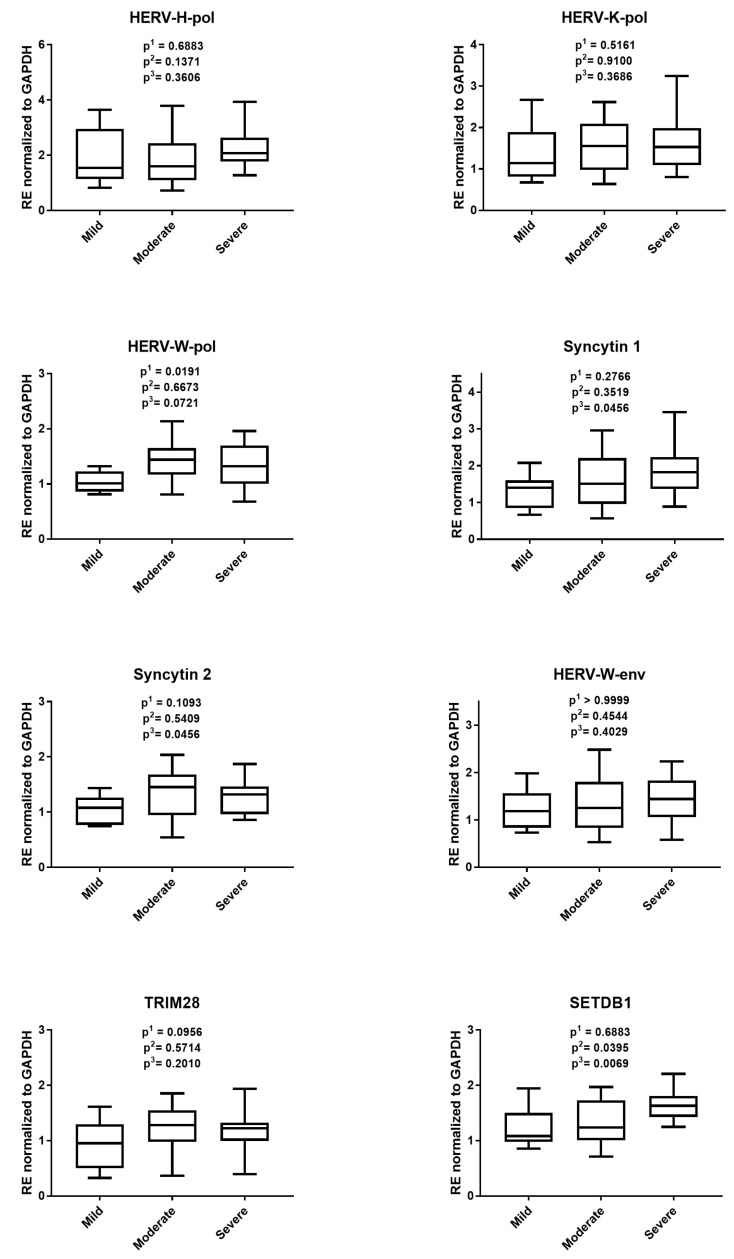
The RNA levels of HERVs, TRIM28, and SETDB1 in 37 patients with diarrhea-predominant irritable bowel syndrome according to the disease activity. RE: relative expression calculated using the 2−∆∆Ct method. Results are represented with whisker box plots: the boxes show the median and interquartile ranges 25–75%, and the whiskers indicate the minimum and maximum values. The *p*-values show the Mann–Whitney test results: p^1^ = mild vs. moderate, p^2^ = moderate vs. severe, p^3^ = mild vs. severe.

As illustrated in Figure 4, the RNA levels of HERVs showed a trend to higher values in patients with more advanced disease states, though mostly without significant differences. The SETDB1 transcript levels were significantly increased in patients with severe disease than in those with mild or moderate disease, while no differences were observed for TRIM28.

## 4. Discussion

The present results document, for the first time, that patients suffering from IBS-D display significantly higher RNA levels of HERV-H-pol, HERV-K-pol, and SYN1, with borderline *p*-values for SYN2 and HERV-W-env, in the whole blood compared to HCs of similar age, while the two groups had comparable levels of HERV-W-pol transcripts. The disease was more prevalent in females than in males. This disparity is consistently observed across distinct populations. The reason is unclear, and it has been ascribed to genetic, hormonal, and immunological differences between the genders [40,41]. Endogenous retroviruses are highly stimulated during intrauterine life, particularly in the placenta and offspring [17,18,42]. However, comparing pregnant women with non-pregnant ones, the former showed lower retroviral transcripts in peripheral blood [43]. This contrasts with the HERV upregulation found in IBS patients. Therefore, it seems unlikely that the higher risk of IBS in women is driven by retroviral mechanisms similar to those that act during pregnancy.

The underlying biological pathways responsible for the aberrant expressions of retroviral elements in IBS-D patients and their clinical meaning remain questionable. A wealth of data evidences that TRIM28 and SETDB1 are involved in HERV silencing. TRIM28 acts as a scaffold protein, recruiting SETDB1 to form a complex with KRAB-ZFPs to inhibit retroviral elements [31,44]. The upregulation of HERVs in our patients cannot, however, be attributed to reduced transcription of TRIM28 or SETDB1, as their expression was normal for the former or even enhanced for the second. In this context, it is worth mentioning that TRIM28 and SETDB1 are crucial for keeping HERVs in a silent state in early embryos and multipotent stem cells [31,44]. In contrast, when distinct somatic cells begin to differentiate, transcription of retroviral sequences is no longer dependent on these repressors [45], which may also function as activators rather than as repressors [46,47]. This could occur in IBS-D patients. Functional interactions between TRIM28/SETDB1 and HERVs might also derive from post-translational effects between their proteins, while we evaluated only their transcriptional profiles. Furthermore, several other genes are presumably involved in the control of HERV transcription, and these may contribute to the aberrant retroviral expression in our patients.

Accumulating findings show enhanced production of inflammatory cytokines and systemic and intestinal low-grade immune activation in IBS-D patients [14,48]. Inflammatory cytokines lead to the proteasome-driven activation of the NF-kB. After its passage into the nucleus, NF-kB binds to specific proviral motives and gives rise to their increased transactivation [49]. HERVs can, in turn, elicit potent inflammatory and immune reactions and evoke pathogenetic actions [20,21,22,23,24,25,26,27]. HERVs can be promoters of neighboring cellular genes [20,21]. Their RNAs, after retro-transcription and reintegration into the DNA, may cause mutations. The recognition of non-self HERV RNAs by TLRs may trigger the stimulation of the inflammasome [23,24,25,26,27]. HERV-K can, for example, activate the NF-kB pathway through TLR8 [50], SYN1 stimulates the TLR3 signal cascade [51], and HERV-W-env influences cytokine production through TLR4 [21]. Furthermore, retroviral antigens can trigger specific and/or cross-reactive antibodies with tissue epitopes [23,52,53]. Enhanced HERV expressions have been documented in several immune-mediated diseases [20,21,22,23,24,25,26,27,28,29]. The consequence may be a vicious circle, resulting in chronic inflammatory and immune responses that may account for the increased membrane permeability in IBS-D patients and hypersensitivity to somatic and visceral stimuli [12].

There is reciprocal interaction between gut microbiota and endogenous retroviruses [54], whose intestinal expression is lost in germ-free mice, while exposure to microorganisms and their products induces retroviral transcription [55]. Both qualitative and quantitative variations of fecal and mucosal gut microbiota have been observed in IBS-D [14]. This dysbiosis of the intestinal microbiome might lead to the HERV overexpression that activates mucosal innate immunity, resulting in increased epithelial permeability, stimulation of nociceptive receptors, and dysregulation of the enteric nervous system [56]. Despite all these findings and considerations, it must be underlined that, as for most diseases associated with enhanced HERV expression, whether their activation is the cause or epiphenomenon of inflammatory and immune reactions remains an intriguing but unsolved dilemma.

It has been suggested that altered epigenetic processes due to environmental factors may contribute to the development of IBS-D [57]. Also, in this case, doubts arise whether epigenetic changes are primary or secondary events. SETDB1 is directly involved in the regulation of epigenetic mechanisms; its increased transcription in IBS patients is the first molecular alteration demonstrated in these subjects. SETDB1 affects a large number of biological functions. It regulates the activation of Th1 genes and the stability of Th2 cells via its effects on retroviruses close to cellular genes of the immune response [33]. Notably, SETDB1 is implicated in the differentiation of gut epithelial cells and participates in the control of bowel inflammation [58,59]. Therefore, the overexpression of SETDB1 in IBS-D patients might mirror its action in driving epigenetic differentiation, expansion, and function of dendritic cells and T cells towards derailed reactivity in individuals with genetic predisposition.

In our study, the RNA levels of HERVs in patients with different disease activity showed a trend, mainly without significant differences, to higher expressions in subjects with more severe disease activity. The SETDB1 transcripts were significantly increased in patients with severe forms of disease compared to those with mild or moderate disease. The fact that the transactivation of SETDB1, and partly the expression of HERVs, were associated with disease activity further supports their contribution to the pathogenesis of the disease.

The global prevalence of IBS in recent years remains doubtful, given the impact of the diagnostic criteria used in assessing the prevalence rates in different studies [2,3]. The burden of IBS is, however, high worldwide. Factors such as changes in lifestyle, dietary habits, and increased stress may contribute to this high prevalence. It is worth mentioning that pollution [60], nutritional changes linked to lifestyle [61], cigarette smoking [62], and intestinal microbiota [54] influence retroviral expression. Therefore, environmental factors, thought to be implicated in the pathogenesis of IBS, could act on targeted biologic systems through HERV- and/or SETDB1-induced effects.

## 5. Conclusions

Based on our results, one wonders whether overexpressions of HERVs and SETDB1 represent easy biomarkers of IBS-D. The highest SETDB1 transcriptional levels in patients with severe forms of the disease may indicate its prognostic significance, leading to patient-tailored treatment. Potentially, several anti-HERV therapeutic measures might be investigated in controlled trials, such as specific anti-RNAs, monoclonal antibodies, cytotoxic T lymphocytes against retroviral epitopes, and antiretroviral treatments [63,64,65], particularly in IBS patients with debilitating disorders or serious impairment of quality of life. For instance, an anti-HERV-W-env humanized monoclonal antibody has been employed in patients affected by multiple sclerosis [63] or type 1 diabetes [66]. Antiretroviral products administered to HIV+ subjects inhibit both HIV viral burden and HERV expression [67,68]. Use of antiretroviral drugs in patients with amyotrophic lateral sclerosis to limit HERV-K upregulation resulted in better disease evolution in those with positive antiviral findings [69]. Administration of a new anti-HIV product [70] to patients with ulcerative colitis in a phase II study provided favorable effects [71]. Proteasome activity is inhibited by antiretroviral drugs [72], with consequent blocking of NF-kB-induced HERV activation [47]. Finally, epigenetic changes are increasingly suggested as potential therapeutic strategies using modulation of the microbiota by various nutrients [73], specific histone deacetylase inhibitors [74], or small molecule compounds [75].

This study has a few limitations. The relatively small sample size may limit the generalizability of the results. The altered expressions of endogenous retroviruses and epigenetic modifiers document systemic biologic effects in IBS patients, in line with extraintestinal symptoms and mood disorders [1,41]. These findings cannot reflect the expression of the same variables in the gastrointestinal mucosa. Peripheral blood samples are minimally invasive and more accessible than intestinal biopsies, which are also not indicated in the current guidelines for IBS patients [5]. In conclusion, taken together, our results suggest that SETDB1 and HERVs might be the main actors in the development of IBS-D and may stimulate research on innovative therapeutic approaches.

## Figures and Tables

**Table 1:** Primers and probes employed to evaluate the transcription levels of HERVs, TRIM28, SETDB1, and GADPH.

Name	Primer/Probe	Sequence
HERV-H pol	Forward	5′-TGGACTGTGCTGCCGCAA-3′
	Reverse	5′-GAAGSTCATCAATATATTGAATAAGGTGAGA-3′
	Probe	6FAM-5′-TTCAGGGACAGCCCTCGTTACTTCAGCCAAGCTC-3′-TAMRA
HERV-K pol	Forward	5′-CCACTGTAGAGCCTCCTAAACCC-3′
	Reverse	5′-TTGGTAGCGGCCACTGATTT-3′
	Probe	6FAM-5′-CCCACACCGGTTTTTCTGTTTTCCAAGTTAA-3′-TAMRA
HERV-W pol	Forward	5′-ACMTGGAYKRTYTTRCCCCAA-3′
	Reverse	5′-GTAAATCATCCACMTAYYGAAGGAYMA-3′
	Probe	6FAM-5′-TYAGGGATAGCCCYCATCTRTTTGGYCAGGCA-3′-TAMRA
Syncytin 1 env	Forward	5′-ACTTTGTCTCTTCCAGAATCG-3′
	Reverse	5′-GCGGTAGATCTTAGTCTTGG-3′
	Probe	6FAM-5′-TGCATCTTGGGCTCCAT-3′-TAMRA
Syncytin 2 env	Forward	5′-GCCTGCAAATAGTCTTCTTT-3′
	Reverse	5′-ATAGGGGCTATTCCCATTAG-3′
	Probe	6FAM- 5′-TGATATCCGCCAGAAACCTCCC-3′-TAMRA
HERV-W env	Forward	5′-CTTCCAGAATTGAAGCTGTAAAGC-3′
	Reverse	5′-GGGTTGTGCAGTTGAGATTTCC-3′
	Probe	6FAM-5′-TTCTTCAAATGGAGCCCCAGATGCAG-3′-TAMRA
TRIM28	Forward	5′-GCCTCTGTGTGAGACCTGTGTAGA-3′
	Reverse	5′-CCAGTAGAGCGCACAGTATGGT-3′
	Probe	6FAM-5′-CGCACCAGCGGGTGAAGTACACC-3′-TAMRA
SETDB1	Forward	5′-GCCGTGACTTCATAGAGGAGTATGT-3′
	Reverse	5′-GCTGGCCACTCTTGAGCAGTA-3′
	Probe	6FAM-5′-TGCCTACCCCAACCGCCCCAT-3′-TAMRA
GAPDH	Forward	5′-CGAGATCCCTCCAAAATCAA-3′
	Reverse	5′-TTCACACCCATGACGAACAT-3′
	Probe	6FAM-5′-TCCAACGCAAAGCAATACATGAAC-3′-TAMRA

**Table 2:** Demographic and clinical characteristics of patients with diarrhea-predominant irritable bowel syndrome (IBS-D) and healthy controls (HCs).

	IBS-D *n* = 37	HC *n* = 95
**Median age** (years) (IQR)	38.5 (25.6–54.7)	36.8 (32.7–45.8)
**Males** n (%)	13 (35)	36 (38)
**Duration of disease** Median (years) (IQR)	1.3 (0.3–5.8)	
**Familiarity** n (%)	7 (20.6)	
**Clinical disease activity**		
Mild n (%)	9 (24.3)	
Moderate n (%)	14 (37.8)	
Severe n (%)	14 (37.8)	

n: number; IQR: interquartile range, expressed as 25 and 75 quartile values.

## Data Availability

The results of this article will be shared at the aggregate/population level upon reasonable request to the corresponding author.
